# Creation of a Pharmacy Student Longitudinal Rotation to Expand the Scope of an Antimicrobial Stewardship Program

**DOI:** 10.3390/pharmacy9030135

**Published:** 2021-08-05

**Authors:** Tiffany Ward, Jaela Fredenrich

**Affiliations:** James A. Haley Veterans’ Hospital, Tampa, FL 33612, USA; Jaela.Fredenrich@va.gov

**Keywords:** pharmacy, antimicrobial stewardship, penicillin allergy

## Abstract

Allergy assessments and penicillin skin testing have emerged as a vital intervention for Antimicrobial Stewardship Programs (ASPs). Investment and involvement in such programs by ASPs, however, are often limited due to resources, time, and personnel constraints. Harnessing an underutilized resource, 4th-year advanced pharmacy practice experience (APPE) students, allows for expanded ASP involvement and scope of practice. We aim to outline and provide insight on how 4th-year APPE students serve as an asset to an ASP. Through our novel longitudinal rotation experience, APPE students complete penicillin allergy assessments, patient education, and work alongside a clinical pharmacist to refer patients for penicillin skin testing if appropriate. Students also achieve many of the education standards required by the Accreditation Counsel for Pharmacy Education (ACPE) for graduation within the Doctor of Pharmacy degree while developing a strong foundation in antimicrobial stewardship and gaining invaluable knowledge for their future. The addition of APPE pharmacy students to our ASP has also enabled our program to achieve its goals and expand involvement and reach within our facility.

## 1. Introduction/Background/Antimicrobial Stewardship Programs

The 2016 Infectious Diseases Society of America (IDSA) and the Society for Healthcare Epidemiology of America (SHEA) recommend that Antimicrobial Stewardship Programs (ASPs) complete allergy assessments and promote penicillin skin testing (PST) for all patients with a history of beta-lactam allergy [1]. More than 10% of patients report having an allergy to penicillin, making it the most common antimicrobial allergy, though less than 1% of these allergies are identified as true IgE-mediated reactions through penicillin skin testing [2]. Patients who report penicillin allergies are known to be exposed to second-line broad-spectrum antimicrobials, have increased prevalence of *C. difficile*, methicillin-resistant *Staphylococcus aureus*, and vancomycin-resistant enterococcal infections, and longer hospital length of stays [2]. Structured allergy assessments have been shown to reduce the above consequences, specifically reducing the use of broad-spectrum antimicrobials, and decreasing hospital length of stay [3]. Penicillin skin testing has been shown to have greater than 97% negative predictive value and has been shown to reduce long-term health care costs [4]. The IDSA and the SHEA recommend that ASPs actively work with allergists to develop testing and treatment strategies at their facility [1].

The Antimicrobial Stewardship Program has been active at the James A. Haley Veterans’ Hospital (JAHVH) for over a decade. The program has a strong foundation in prospective audit and feedback, and significant physician and leadership support. Along with the ID Physician Champion, we have two full-time ID Pharmacists, a PGY-2 Infectious Diseases Pharmacy Resident, and on average four PGY-1 pharmacy residents that rotate through an ID elective. Annually, a progress report is published to share the accomplishments and advancement of the program as well as establish new goals for the future. In 2017, our annual goals included expanding the scope of the ASP by developing a structured antibiotic allergy assessment and expanding access to PST to qualifying patients. Two quality improvement projects were completed which demonstrated several areas for ASP involvement. First, we reviewed the accuracy and completeness of allergy documentation, and found that structured allergy assessments were only completed 14% of the time, with most penicillin allergies failing to include a description of the reaction. Secondly, by specifically reviewing use of benzylpenicilloyl polylysine (Pre-Pen^®^) in our Allergy Clinic, we determined that our overall rate of PST was rather low, and more pointedly, penicillin allergy labels were remaining in the patient’s record even after a negative skin test.

Collaboration with our Allergy and Immunology service resulted in increased ASP oversight to enhance patient awareness and referrals to Allergy clinic for PST. Education for our Allergy and Immunology colleagues was completed to emphasize the importance of removing an allergy after a negative skin test.

A major challenge of expanding the scope of our program to achieve our allergy assessment and PST goals was resources, time, and personnel. However, by harnessing an underutilized resource, 4th-year advanced pharmacy experience (APPE) student and rotation, and engineering a new longitudinal rotation structure, we could successfully take on this challenge.

## 2. Involving Pharmacy Students in the Antimicrobial Stewardship Program

The national emphasis on antimicrobial stewardship suggests that doctoral pharmacy programs should incorporate antimicrobial stewardship training into the curriculum. The Accreditation Counsel for Pharmacy Education (ACPE) sets the accreditation standards and key elements for the professional program in pharmacy leading to the Doctor of Pharmacy degree. Key elements of current education standards focus on developing a knowledge of the foundational sciences, patient-centered care, medication system management, interprofessional collaboration, leadership, and professionalism. These key elements are to be achieved through a mix of didactic curriculum and hands-on experience. While infectious diseases and antimicrobial stewardship are not primary subjects, these are addressed during lectures spanning microbiology, pharmaceutics, pharmacokinetics, pharmacology, and epidemiology. Hands-on experience is to be gained through advanced pharmacy practice experiences (APPEs) in four practice settings: community, ambulatory patient care, hospital/health system pharmacy, and inpatient general medicine. This is completed over the course of 36 weeks and totals 1440 h. Antimicrobial stewardship and infectious diseases are considered elective rotations under the current standard [4].

As antimicrobial stewardship is not a required part of the current ACPE educational standard for Doctor of Pharmacy candidates, students may graduate pharmacy programs without the foundational knowledge of how to participate in an ASP [5]. However, APPE students should have acquired the foundational clinical knowledge to assess the appropriateness of antimicrobial use, monitor patients for safety and efficacy, assess duration of antimicrobial therapy, make recommendations to the health care team to optimize antimicrobial therapy, and provide education to patients and health care professionals about infectious diseases [6]. The American Society of Health-System Pharmacists (ASHP) recognizes that pharmacists serve a prominent role in ASPs and that training opportunities in antimicrobial stewardship are limited. Expanding ASP training opportunities for pharmacists and pharmacy students is a goal for ASHP [7]. Involving students in antimicrobial stewardship programs during APPE rotations is a tangible step preceptors can take in building this foundation and increasing the number of pharmacists with antimicrobial stewardship training.

## 3. Developing a Longitudinal Pharmacy Student Rotation

### 3.1. Rotation Model

Beginning in May 2018, JAHVH began offering block rotations for our APPE pharmacy students. The block rotation program was designed to expose students to multiple clinical practice areas of the Veterans’ Administration (VA) facility, allow students to connect with multiple preceptors and other students, as well as provide students the opportunity to complete a longitudinal project or activity, all while reducing administrative and onboarding barriers. The block consists of 3 separate six-week-long APPE rotations, with options for over 15 unique rotations amongst 50 clinical preceptors. On average, our APPE program hosts 8 students per block, with 3 blocks per year. During each block, one APPE student will be assigned an individual longitudinal activity. As such, our ASP sponsored a longitudinal rotation designed to have students interview patients admitted to our facility that had a documented allergy to penicillin. Through these patient interviews, students collect information about the patient’s allergies, determine whether the patient is a candidate for PST or penicillin oral challenge, educate the patient about penicillin allergies and skin testing, document their interventions in the electronic medical record (EMR), and update patient allergy information. The overarching goal of the rotation was to improve antimicrobial allergy documentation and increase access to PST as a means to achieve our annual ASP goal and to introduce APPE students to the breadth and scope of an antimicrobial stewardship program. Most notably, the student does not have to be assigned to an infectious diseases APPE rotation to participate in the PST longitudinal rotation.

The longitudinal rotation spans the entirety of their APPE block, 18 weeks. The rationale for conducting the rotational experience over an extended duration of time is to allocate students small segments of time to focus on their longitudinal project while on their required APPE rotations. For our particular longitudinal rotation, students have the opportunity to complete their responsibilities in either a 30–60 min daily segment or in a 3–4 h weekly segment. This rotation style provides the opportunity for APPE students to work on their time management, oral and written communication, and patient education skills. The longitudinal rotation format is also preferrable for preceptors, who have competing priorities such as other APPE students, pharmacy residents, administrative responsibilities, and patient care responsibilities.

### 3.2. Training Students

**Figure 1:**
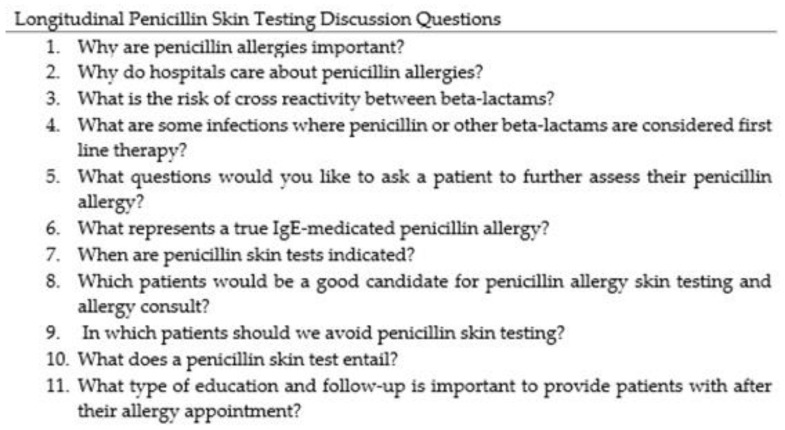
Penicillin Allergy Training Discussion Questions.

To ensure that our students were able to independently identify patients that are appropriate for interview, recognize which patients are candidates for PST, and appropriately document their interventions in the EMR, we developed a complete and concise training program. First, we organized a reading list of recent high-impact penicillin allergy publications for review [8,9,10,11,12]. These readings are expected to be completed during their first week of the longitudinal rotation. In addition, there are YouTube™ videos on PST, and patient education handouts to review. During the second week of the longitudinal rotation, there is an informal topic discussion to assess the student’s level of understanding of penicillin allergies and recognizing patients that are candidates for PST. A list of formal questions was developed to assess the students baseline knowledge and understanding of the introductory readings (Figure 1). It is left to the preceptor’s discretion to determine whether students need additional training or discussion prior to proceeding to the next step of training, hands-on training.

At the start of hands-on training, students undergo a one-hour orientation session that includes Theradoc™ training [our facility utilizes Theradoc™, a clinical decision support software, for ASP interventions and tracking], an EMR overview, and training for documenting their interventions in the EMR and Theradoc™. This training may be conducted by the preceptor or by the previous APPE students. Students will then work side by side with the preceptor to identify and assess whether a patient is a candidate for a penicillin allergy interview.

**Figure 2:**
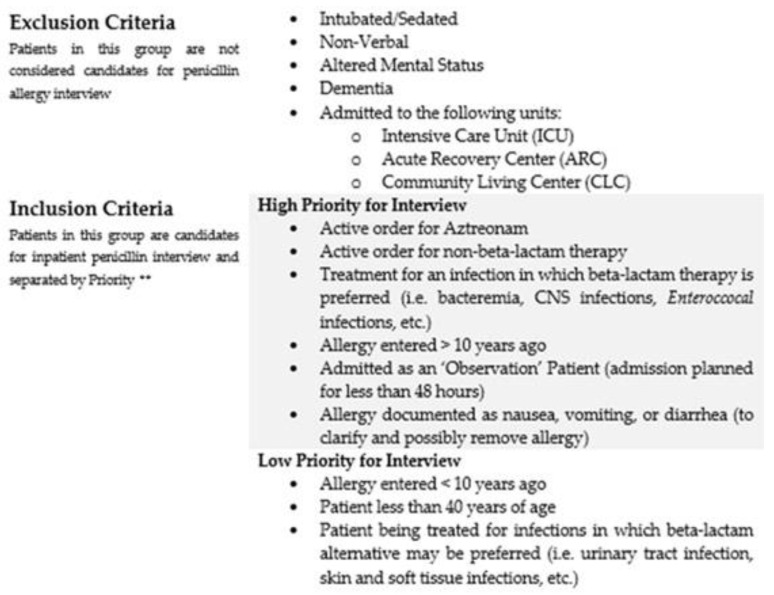
Algorithm of Chart Review. ** Only one criterion is required to be considered high priority for interview.

When designing our longitudinal rotation experience, we developed a ‘Pharmacy Assistant Alert’ in Theradoc™ for patients admitted to our facility with a penicillin allergy. The alert identifies the patient, inpatient unit location, and the medication allergy. Students are expected to access the report at least weekly and identify patients that are candidates for penicillin allergy interview. To help students prioritize patients identified on the penicillin allergy report, we developed an ‘algorithm of chart review’ (Figure 2). The algorithm is based on current literature about patients that are candidates for PST, as well as expert opinion from the Allergy and Immunology colleagues at our facility. The algorithm considers a patient’s time since allergy documentation, admitting team/unit, discharge status, admission diagnosis, current antimicrobial use, and local PST experience.

**Figure 3:**
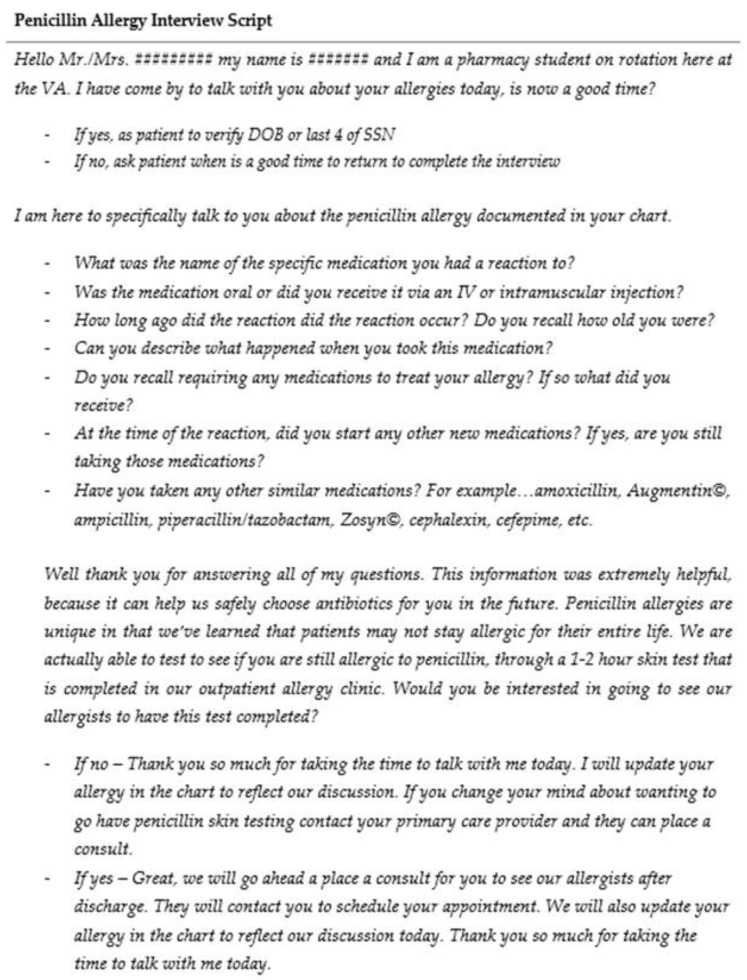
Penicillin Allergy Interview Script.

Once a patient is identified, the students will shadow a preceptor for a penicillin allergy interview and see how the preceptor makes recommendations for PST, if appropriate. We developed a formal interview script (Figure 3) for students to utilize during patient interview. The script was developed to ensure students ask all pertinent questions and provide appropriate education to patients while working independently. Students shadow the preceptor for one to two patient interviews, after which the student and preceptor switch roles and the student leads the interview. It is up to preceptor discretion to allow students to start interviewing patients independently; this takes one to five interviews on average.

All resources mentioned above were combined into a formal rotation syllabus (Supplementary Syllabus) that also outlines the student’s responsibilities, expectations, and rotation timeline. It is anticipated that it will take approximately 4 weeks before the student will be interviewing patients independently. Students should therefore be conducting independent interviews and assessments for 14 out of 18 weeks of the longitudinal rotation.

### 3.3. Student Activities

#### 3.3.1. Patient Interview and Education

Patient interview and education serve as the core of this rotation. To improve allergy documentation in the EMR, more comprehensive patient allergy interviews must be conducted. Students use their clinical reasoning and problem-solving skills to determine whether a patient is a candidate for PST, based on their assessment of the patient’s allergy description and history. If a patient is determined to be eligible for PST, students utilize a shared decision-making approach to offer referral to the Allergy and Immunology clinic for further assessment and possible PST or oral challenge. Students are to provide patients with the one-page CDC “Is it really a penicillin allergy?” handout at the end of their allergy interview. These patient interview skills touch on standards 1–4 of the ACPE educational standards; specifically targeting patient-centered care, foundational knowledge and problem solving, education, and professionalism [4]. If the patient is not determined to be eligible for PST, the student will review the information collected with the preceptor, and in the majority of cases, the EMR documentation can still be updated with more relevant history and recommendations to continue avoiding certain antibiotics.

#### 3.3.2. Intervention Documentation

Following patient interview and education, students are required to document their interventions in Theradoc™ and the EMR. Students utilize a note template to document their interventions in the EMR. The note template follows hospital regulations for student notes and includes references to support our recommendations. Students are responsible for documenting the details of their patient interview and documenting their assessment of the patient’s penicillin allergy, and the results of their shared decision-making discussion. Students also log their activity in Theradoc™; this documentation includes selecting if they spoke with the patient, wrote a note and updated the allergy, if the patient was a candidate for PST, and if the patient accepted or declined referral to the Allergy and Immunology clinic, if applicable. The documentation is sent to the preceptor for review prior to signing in the EMR. The preceptor is responsible for reviewing the note and providing feedback to the APPE student. The preceptor is also responsible for updating the patient’s allergy in the EMR and entering the formal consult to Allergy and Immunology service, if applicable. If antimicrobial therapy changes are appropriate, based on patient interview, the preceptor will reach out to the inpatient care team or physician to make therapy recommendations. The preceptor will additionally alert the inpatient care team or physician to the signed allergy assessment note for review.

#### 3.3.3. Precepting

There are opportunities during the longitudinal experience where APPE students overlap. If overlap between students occurs, typically during the last 3 to 4 weeks of the 18 week rotation, students are given the opportunity to act as the next longitudinal student preceptor and train the next student to conduct interviews and document interventions. Students that opt to precept are given the opportunity to direct the one-hour orientation session and hands-on training. It is up to preceptor discretion to determine whether the student is performing at a sufficient level to precept other students. If the preceptor determines the student should not precept another student or the student is not interested in precepting, the one-hour orientation session and hands-on training will be conducted by the preceptor. We recommend that the preceptor remain the primary preceptor for the didactic portion of the training.

### 3.4. Student Feedback

Students are encouraged to employ self-evaluation techniques throughout their longitudinal rotation. Students meet with their preceptor as needed throughout the 18 week longitudinal rotation. Primary reasons for meeting include orientation and training, questions about a specific patient, feedback on notes, and formal feedback every 6 weeks, otherwise the student is expected to work independently. The autonomy provided to students prepares them for their future as pharmacists with less oversight by supervisors, and therefore students are encouraged to find ways to evaluate their own performance. During formal feedback sessions every 6 weeks, preceptors listen to the student’s self-evaluation and provide more formal feedback on their performance. Students are also provided with a formal report on their interventions completed in Theradoc™. These reports provide students with feedback on the number of interventions conducted per week, how many are candidates for PST, and how many accept a referral to clinic.

All training documents and templates are uploaded in a shared drive and in an editable format; students are encouraged to update and add to all documents as they see fit to help continuously improve the rotation and training materials. We have encouraged the students to create and maintain a “FAQ” document of questions that patients ask during their interviews, so each new APPE student is prepared.

## 4. Rotation Outcomes and Lessons Learned

**Figure 4:**
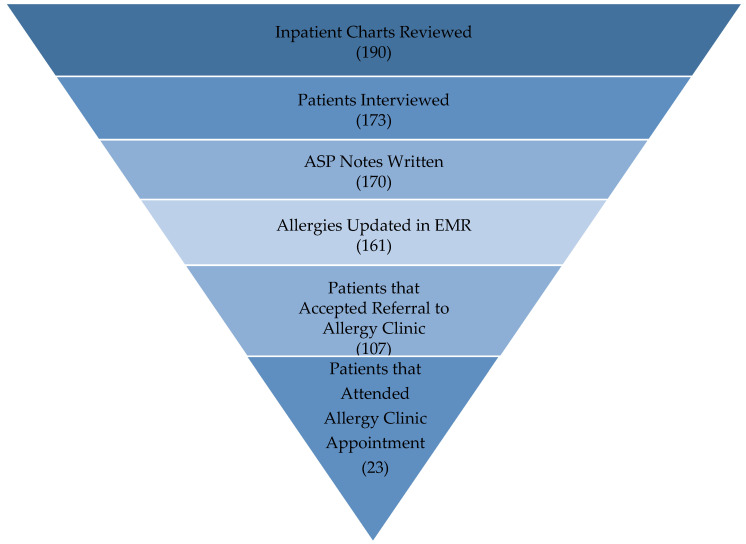
Student Interventions from May 2018 to March 2021. The number in parenthesis (*N*) represents the number of patients for this step of the penicillin allergy assessment and skin testing process.

As of 1 March 2021, our longitudinal students have interviewed 190 patients, made formal written recommendations for PST for 170 patients, and updated allergies in the EMR for 161 patients. One hundred and seven patients accepted a referral to the Allergy and Immunology Clinic; however, only 23 patients attended their appointment (Figure 4).

The number of patients scheduled for PST has continued to increase annually at our facility since the initiation of our rotation. Prior to the initiation of our longitudinal rotation, a maximum of 33 patients were scheduled for penicillin skin testing at our facility. In 2018, the year our students became involved, 38 patients were scheduled for PST which increased to 47 patients in 2019. With the COVID-19 pandemic, we have noticed that the number of patients scheduled for PST decreased to 40 patients in 2020. COVID-19 had minimal impact on the day to day activities of our longitudinal rotation; the only change being that students are conducting patient interviews via telephone rather than face-to-face. The COVID-19 pandemic had a more significant impact on the Allergy and Immunology clinic. At the end of March 2020, all elective procedures and clinic appointments, including PST, were postponed and resumed in early August 2020. Despite four months of clinic closure, 40 patients were scheduled for PST in 2020. Thirty patients underwent PST, with 23 patients having a negative skin test and all but 1 patient had their allergy removed from the EMR after a negative penicillin skin test. Since the initiation of this longitudinal project, we also continue to see improvement in the number of patients for which the allergy was removed after a negative penicillin skin test (Table 1).

### 4.1. Successes

Longitudinal rotation experiences have increased student engagement at our facility; feedback from students about the PST longitudinal program has been overwhelmingly positive. Since the beginning of this longitudinal rotation in May 2018, eight students have rotated through. Students report appreciating the independence they have on the rotation and being able to work on their own schedule at their own pace. Other students have reported that they have really liked being part of the antimicrobial stewardship team and learning about a specific ASP intervention. Students repeatedly point out improvements in their communication skills, writing skills, and time management skills, which are part of the core educations standards established by the ACPE [4]. The longitudinal experience is designed to optimize student engagement by allowing students to work on a project in addition to their primary rotation responsibilities. Students are offered the opportunity to present the PST data as a poster for our patient safety week, antibiotic awareness week, or at ASHP Midyear. In previous years, students have also been given the opportunity to shadow a penicillin skin test in the Allergy and Immunology Clinic; this experience has been limited in 2020 due to the COVID-19 pandemic. Students have reported that they enjoy the penicillin skin test shadowing experience as this increased their confidence when talking to patients about the procedure.

This longitudinal experience increased interdisciplinary engagement within the antimicrobial stewardship team. This longitudinal rotation created a synergistic relationship with the Allergy and Immunology service; we have achieved one of ASP’s goals and increased consults for our Allergy and Immunology colleagues. As our ASP continues to expand its scope and reach within the hospital, we continue to develop stronger relationships with our pharmacy management, who continue to provide resources for ASP growth.

Interviewing patients and documenting interventions is a great way to showcase the workload of the ASP. Each of these activities is an encounterable intervention that is included in the daily workload of an ASP program. Most importantly, conducting patient interviews has improved allergy documentation at our facility. Having more accurately documented antimicrobial allergies has enabled health care professionals to make better antimicrobial recommendations for patients with documented penicillin allergies. We have also anecdotally noticed that primary care providers, hospitalists, and other pharmacists have improved their allergy interviews and documentation now that our ASP is making these interventions.

### 4.2. Challenges and Future Implications

As with all projects, we have experienced challenges. The first hurdle we had to overcome is level of student knowledge and motivation. This longitudinal experience is an extra requirement in addition to their primary rotations and students are not given a formal grade for this experience, and therefore motivation can be lacking for some students. It is up to the discretion of the longitudinal preceptor to provide feedback to the primary APPE rotation preceptor to be included in formal rotation evaluations. Because students are expected to work independently, the success of this project is dependent on the student’s level of knowledge and motivation. Depending on the level of baseline knowledge and student engagement, the workload may vary month to month. It is important that as preceptors we connect their work on the rotation to meaningful patient outcomes. When students have the opportunity to see a patient complete skin testing and have an outdated allergy removed from their record, the student can actualize the value of their work.

Remote allergy removal, the removal of allergies from other health care facilities, has also been a major challenge. After a negative penicillin skin test, the penicillin allergy is removed from the EMR at our facility, patients are educated that their test was negative, and that they are no longer allergic to penicillin. It is then left to the patient to contact every medical facility and physician they have seen to also remove their penicillin allergy. It is impossible to verify whether this is carried out in the civilian setting. However, we can see remote penicillin allergies across all VA systems. It is imperative to remove these remote penicillin allergies. Often, patients that had a negative penicillin skin test will have their penicillin allergy removed from our facility but not at other VA facilities, resulting in the penicillin allergy being re-entered into the EMR on future encounters. To remedy this issue, the ASP pharmacist at our facility reaches out to each remote site individually to inform them of the negative penicillin skin test to remove the allergy [13,14]. This is carried out on a quarterly basis.

Our most significant challenge has been ensuring patient follow-up. Over 100 patients accepted referral to the allergy clinic during their hospital admission, but less than 25% of patients schedule or show up to their appointment. This provides strong supporting evidence for initiating an inpatient PST program at our facility. At our facility, pharmacists are unable to conduct PST, and therefore we continue to work with our Allergy and Immunology and ID colleagues to establish an inpatient PST program. Until an inpatient PST program is established, we have started prioritizing our patient interviews based on discharge status by targeting patients closer to discharge and placing Allergy and Immunology consults at the time of discharge to increase patient compliance with scheduling appointments.

## 5. Conclusions

Utilizing pharmacy students as members of the antimicrobial stewardship team is an effective strategy to expand the scope of the program and the skillset of the students. Pharmacy students are equipped with foundational clinical infectious diseases knowledge, and can become an asset with structured training and longitudinal exposure, ultimately benefiting from stewardship experience prior to graduation. We encourage other facilities to train and utilize APPE pharmacy students on their antimicrobial stewardship teams and establish an allergy assessment and penicillin skin testing program if resources are available.

## Figures and Tables

**Table 1:** Annual Facility Penicillin Skin Testing Clinic Results.

	2015	2016	2017	2018	2019	2020
**Test Scheduled**	17	14	33	38	47	40
**Test Conducted** **(% of Tests Scheduled)**	11 (65%)	14 (100%)	30 (91%)	36 (95%)	44 (94%)	30 (75%)
**Negative Test** **(% Tests Conducted)**	9 (82%)	12 (85%)	19 (63%)	32 (89%)	42 (95%)	23 (76%)
**Positive Reaction**	2	2	11	4	2	7
**Allergy Removed with Negative Test** **(% of Negative Tests)**	4 (44%)	2 (17%)	10 (53%)	25 (78%)	38 (90%)	22 (95%)
**Remote Allergy Still Exists at Other VA Facility**	1	6	7	15	11	16

Note that student involvement began in May 2018. Prior to May 2018, consults to the allergy clinic were primarily from primary care.

## Supplementary Materials

The following are available online at https://www.mdpi.com/article/10.3390/pharmacy9030135/s1, Supplementary Syllabus: Penicillin Skin Testing Longitudinal Rotation Syllabus.

## References

[1] Barlam T.F., Cosgrove S.E., Abbo L.M., MacDougall C., Schuetz A.N., Septimus E.J., Srinivasan A., Dellit T.H., Falck-Ytter Y.T., Fishman N.O. (2016). Implementing an antibiotic stewardship program: Guidelines by the Infectious Diseases Society of America and the Society for Healthcare Epidemiology of America. Infect. Dis..

[2] Macy E., Contreras R. (2014). Health care use and serious infection prevalence associated with penicillin “allergy” in hospitalized patients: A cohort study. J. Allergy Clin. Immunol..

[3] Unger N.R., Gauthier T.P., Cheung L.W. (2013). Penicillin skin testing: Potential implications for antimicrobial stewardship. Pharmacotherapy.

[4] Accreditation Council for Pharmacy Education Accreditation Standards and Guidelines for the Professional Program in Pharmacy Leading to the Doctor of Pharmady Degree. Guidelines Version 2.0. https://www.acpe-accredit.org/pdf/S2007Guidelines2.0_ChangesIdentitiedInRed.pdf.

[5] Hidayat L., Patel S., Veltri K. (2012). Active-learning implementation in an advanced elective course on infectious diseases. Am. J. Pharm. Educ..

[6] American Association of Colleges of Pharmacy Center for the Advancement of Pharmacy Education. Education Outcomes 2013..

[7] ASHP (2010). Statement on the pharmacist’s role in antimicrobial stewardship and infection prevention and control. Am. J. Health Syst. Pharm..

[8] Evaluation and Diagnosis of Penicillin Allergy for Healthcare Professionals. https://www.cdc.gov/antibiotic-use/community/for-hcp/Pencillin-allergy.html.

[9] Valsman A., McCready J., Powls J. (2017). Clarifying a “Penicillin” Allergy A Teachable Moment. JAMA.

[10] Trubiano J.A., Adkinson N.F., Phillips E.J. (2017). Penicillin allergy is not necessarily forever. JAMA.

[11] Chen J.R., Khan D.A. (2017). Evaluation of Penicillin Allergy in the Hospitalized Patient: Opportunities for Antimicrobial Stewardship. Curr. Allergy Asthma Rep..

[12] Jones B.M., Bland C.M. (2017). Penicillin skin testing as an antimicrobial stewardship initiative. Am. J. Health Syst. Pharm..

[13] Devchand M., Kirkpatrick C., Stevenson W., Garrett K., Perera D., Khumra S., Urbancic K., Grayson M.L., Trubiano J.A. (2019). Evaluation of a pharmacist-led penicillin allergy de-labelling ward round: A novel antimicrobial stewardship intervention. J. Amtimicrob. Chemother..

[14] Stone C.A., Trubiano J., Coleman D.T., Rukasin C., Phillips E.J. (2020). The Challenge of De-Labeling Penicillin Allergy. Allergy.

