# Supplementary material for: Creation of a Pharmacy Student Longitudinal Rotation to Expand the Scope of an Antimicrobial Stewardship Program
Source: Pharmacy (Basel). 2021 Aug 5;9(3):135. doi: 10.3390/pharmacy9030135 (PMC8396317; doi:10.3390/pharmacy9030135)
Supplement: Supplementary file 1 [file pharmacy-09-00135-s001.zip › pharmacy-1288257-SI.pdf]

Supplementary Syllabus: Penicillin Skin Testing Longitudinal Rotation Syllabus

LONGITUDINAL EXPERIENCE: Penicillin Skin Testing Referral

PRECEPTOR: Tiffany Ward (Tiffany.Ward2@va.gov) 813-972-2000 x5436 OR 407-970-7053 (Cell)

DEFINITION/IMPORTANCE: More than 10% of the JAHVA patient population have a documented penicillin allergy, but less than 1% are likely to be truly allergic. This activity will help facilitate review of penicillin allergies during an inpatient admission or outpatient appointment and coordinate referral to outpatient Allergy Clinic for possible penicillin skin testing. This promotes antimicrobial stewardship principles.

OBJECTIVES: To be able (1) to review and evaluate a documented penicillin allergy, (2) to interview a patient, (3) determine candidacy for penicillin skin testing, and (4) to effectively communicate verbally and written, a referral to Allergy Clinic for skin testing.

RESPONSIBILITIES: Activities may include but are not limited to:

- ❖ Utilize daily report in Theradoc to identify new admitted patients with a penicillin allergy
- ❖ Perform CPRS Chart review to gather and evaluate a patient's medical and medication history. Follow Algorithm in Appendix I.
- ❖ Interview patient to clarify information regarding allergy if necessary.
  - ○ Provide education on benefit of Penicillin Skin Testing
  - ○ Briefly review process of skin testing
  - ○ Make offer for referral to Allergy Clinic
- ❖ Document outcome of activities in Theradoc Interventions
- ❖ If offer for penicillin skin testing is accepted by patient, write a CPRS chart note using assigned template. Notes should be written same day as patient interview. Preceptor will place consult to Outpatient Allergy Clinic.
- ❖ Use Theradoc Intervention Reporting to create summary reports on outcomes of activities every 6 weeks (end of each rotation).

EXPECTATIONS: The time commitment and schedule of this activity is flexible and can be agreed upon with the primary rotation preceptor and may change with each rotation.

- ❖ Daily activities should not exceed 1 hour and can be completed at any time during the day.
- ❖ Attempt to interview at least 3 patients per week
  - o Complete at least 3-5 chart notes per week
- ❖ Email or Skype check-in with ID preceptor every week on progress, issues, or challenges.

Week 1: Review Background readings and discuss with preceptor. Introduction to Theradoc. Shadow an encounter.

Week 2: Practice in Theradoc. Review chart work-up with preceptor. Continue to shadow and by end of week, perform an observed interview, and write at least one note.

Week 3: At least 3 patient interviews will be observed, with accompanied notes. Skills check-off. Document interventions in Theradoc.

Week 4 & onward: Perform activities independently

Week 6: Meet with preceptor to review Intervention Reporting in Theradoc for Progress Report

Week 12: Progress Report

Week 18: Final Progress Report

**In January, April, July, and October, the student will also have opportunity to review quarterly Stewardship reports of Penicillin Skin Testing outcomes in clinic in order to evaluate how many patients attended the appointments and were successfully tested.*

BACKGROUND READING
(located in S:\Pharmacy\Clinical\Students\Longitudinal Study) :

1. Evaluation and Diagnosis of Penicillin Allergy for Healthcare Professionals
   https://www.cdc.gov/antibiotic-use/community/for-hcp/Penicillin-Allergy.html
2. "*Clarifying a Penicillin Allergy*" A teachable moment. JAMA 2017
3. Trubiano JA, Adkinson NF, and Phillips EJ. Penicillin allergy is not necessarily forever. *Jama 2017. 318:* 82-83.
4. Chen JR, and Khan DA. Evaluation of Penicillin Allergy in the Hospitalized Patient: Opportunities for Antimicrobial Stewardship. *Current Allergy and Asthma Reports 2017*, *17*: 40.
5. Barlam TF, et al. Implementing an antibiotic stewardship program: guidelines by the Infectious Diseases Society of America and the Society for Healthcare Epidemiology of America. *Clinical Infectious Diseases 2016; 62*: e51-e77. Section on Penicillin Skin Testing
6. Jones BM, and Bland CM. Penicillin skin testing as an antimicrobial stewardship initiative. *Am J Health-Syst Pharm.* 2017; 74:232-7.

7. Pre-Pen® Website: https://penallergytest.com/
   Video on Skin Test: https://www.youtube.com/watch?v=Ie3oiT39s48
8. JAHVA Pharmacy MUE
   a. Penicillin Skin Testing
   b. Allergy Documentation

Materials:

Appendix I – Algorithm of Chart Review (Inclusions/Exclusions of who to interview)/Med Rec Questions

- Exclusion Criteria: The following patients are not considered candidates for penicillin allergy interview:
  - Intubated/Sedated
  - Non-verbal
  - Altered Mental Status/Dementia
  - Admitted to one of the following units
    - Intensive Care Unit (ICU)
    - Acute Recovery Center (ARC)
    - Community Living Center (CLC)
- Inclusion Criteria: The following patients are candidates for inpatient penicillin skin testing interview and separated by priority of interview:
  - High Priority Interview Candidate:
    - Active order for aztreonam
    - Active order for non-beta-lactam therapy
    - Being treated for and infection in which beta-lactam therapy is preferred, i.e. bacteremia, CNS infections, *Enterococcal infections*, etc.
    - Allergy entered > 10 years ago
    - Admitted as an 'Obs' patient (Medicine Observation or Surgery Observation)
    - Allergy documented as nausea, vomiting, diarrhea, etc. (to clarify and possibly remove allergy – would not need skin testing)
  - Low Priority Interview Candidate: Interview during admission if time
    - Observed allergy entered in the last 5-10 years
    - Patients < 40 years of age
    - Patient being treated for mild infections or infections in which beta-lactam alternatives may be preferred, i.e urinary tract infections, skin and soft tissue infections, etc.

Patient Handouts - (located in S:\Pharmacy\Clinical\Students\Longitudinal Study)

Script

- Hello Mr/Mrs. ######### my name is ####### and I am a pharmacy student on rotation here at the VA. I have come by to talk with you about your allergies today, is now a good time?

    - If yes, as patient to verify DOB or last 4 of SSN
    - If no, ask patient when is a good time to return to complete the interview
- I am here to specifically talk to you about the penicillin allergy documented in your chart.
    - What was the name of the specific medication you had a reaction to?
        - Was the medication oral or did you receive it via an IV or intramuscular injection?
    - How long ago did the reaction did the reaction occur? Do you recall how old you were?
    - Can you describe what happened when you took this medication?
    - Do you recall requiring any medications to treat your allergy? If so what did you receive?
    - At the time of the reaction, did you start any other new medications?
        - If yes, are you still taking those medications?
    - Have you taken any other similar medications? For example, amoxicillin, Augmentin, ampicillin, piperacillin/tazobactam, Zosyn, cephalexin, cefepime, etc.
- Well thank you for answering all of my questions. This information was extremely helpful, because it can help us safely choose antibiotics for you in the future. Penicillin allergies are unique in that we've learned that patients may not stay allergic for their entire life.
- It is important to know this information, as having a penicillin allergy means you might not always receive the best antibiotic when you have an infection. You may get a more expensive antibiotic, a more broad-spectrum antibiotic (meaning it kills more bacteria than just the one causing the infection). Some of these antibiotics can have more side effects, and they can put you at increased risk for more resistant infections in the future.
- We are actually able to test to see if you are still allergic to penicillin, through a 1-2-hour skin test that is completed in our outpatient allergy clinic. Would you be interested in going to see our allergists to have this test completed?
- [Provide details about skin testing if patients asks]
    - If no – Thank you so much for taking the time to talk with me today. I will update your allergy in the chart to reflect our discussion. If you change your mind about wanting to go have penicillin skin testing contact your primary care provider and they can place a consult.
    - If yes – Great, we will go ahead a place a consult for you to see our allergists after discharge. They will contact you to schedule your appointment. We will also update your allergy in the chart to reflect our discussion today. Thank you so much for taking the time to talk with me today.

** Only IgE type reactions are beneficial to confirm/deny with skin testing ** Other "allergies" or non-specific reactions, such as drowsiness, seizures, elevated liver enzymes, etc – are not IgE allergic reactions and therefore skin testing would not be useful. We can still interview these patients to clarify information and update their allergy if needed, but we likely should not offer skin testing. When in doubt, ask the preceptor prior to interviewing the patient.

CPRS note Template

- Spoke to patient
- Unable to speak to patient

Appendix II – Theradoc Tutorial

Website: https://vhatamapp1tdoc.v08.med.va.gov/theradoc/login/
